# Demographics and Personality Factors Associated with Burnout among Nurses in a Singapore Tertiary Hospital

**DOI:** 10.1155/2016/6960184

**Published:** 2016-07-12

**Authors:** Shin Yuh Ang, Satvinder S. Dhaliwal, Tracy Carol Ayre, Thendral Uthaman, Kuan Yok Fong, Choo Eng Tien, Huaqiong Zhou, Phillip Della

**Affiliations:** ^1^Nursing Division, Singapore General Hospital, Bowyer Block, 11 Third Hospital Avenue, Singapore 168751; ^2^School of Nursing, Midwifery and Paramedicine, Curtin University, Building 405, Kent Street, Bentley, WA 6102, Australia

## Abstract

*Background*. The aim of the study was to evaluate the prevalence and extent of burnout among nurses in Singapore and investigate the influence of demographic factors and personal characteristics on the burnout syndrome.* Methods*. A cross-sectional survey design was adopted. All registered nurses working in Singapore General Hospital were approached to participate. A questionnaire eliciting data on demographics, burnout (measured using the Maslach Burnout Inventory, MBI), and personality profile (measured using the NEO Five-Factor Inventory, NEO-FFI) was used.* Results*. 1830 nurses out of 3588 responded (response rate: 51%). Results from 1826 respondents were available for analysis. The MBI identified 39% to have high emotional exhaustion (EE, cut-off score of >27), 40% having high depersonalization (DP, cut-off score of >10), and 59% having low personal accomplishment (PA, cut-off score of <33). In multivariable analysis, age, job grade, and neuroticism were significantly associated with each of the 3 components of the MBI. Staff nurses less than 30 years with high to very high neuroticism were more likely to experience high EE, high DP, and low PA.* Conclusion*. Younger nurses in Singapore are at increased risk of burnout. Personality traits also played a significant role in the experience of burnout.

## 1. Background

A rapidly ageing population, coupled with an expanding resident population, has led to an increased demand for health care services in Singapore. By year 2020, there will be an additional 2200 beds in acute hospitals and 1900 beds in community hospitals. In addition, services in the primary and long term care sectors will also be enhanced [1]. The expansion of services entails that, in the near future, Singapore will face an even more acute shortage of nurses.

It is well-recognised that a shortage of registered nurses leads to a poorer quality of care. In a meta-analysis conducted by the Agency for Healthcare Research and Quality [2], it was concluded that although the association was not necessarily causal, increased nurse staffing in hospitals was associated with lower hospital-related mortality, lower rate of failure to rescue, cardiac arrest, hospital acquired pneumonia, and other adverse events.

Although, in recent years, there were increased efforts in Singapore to train as well as recruit nurses from overseas, equally important is the need to retain nurses within the profession. It is well-recognised that reducing attrition is an ongoing challenge. To intensify the problem, more newly qualified nurses are leaving the profession. To illustrate, it was reported that 13% of newly qualified nurses had changed principal jobs after one year, and 37% expressed being ready to change jobs [3].

Nurses in Singapore are in the forefront of rapid changes in our health care system. These include faster turnover of patients, adoption of new sophisticated technology, and need for integrated care as well as renewed emphasis on productivity. Nursing is known to be a physically and mentally demanding profession, and nurses are known to be at increased risk of burnout which in turn is known to be a risk factor for nurses' intention to quit the job [4].

Given shortage of nurses and increased demand for health care services, it is timely to evaluate the prevalence of burnout among nurses in Singapore and investigate the influence of demographic and personality variables on the experience of burnout.

### 1.1. Burnout

Burnout is commonly defined as a syndrome of feelings of emotional exhaustion, depersonalization, and reduced personal accomplishment. The professional feels fatigue and is unable to provide basic care or form a caring relationship with his/her patients [5].

The burnout syndrome has been widely studied as it is deemed to be a major modifiable factor in improving the working conditions of professional staff involved in human service. A better understanding of the influencing factors for nurse burnout will enable hospital administrators to identify promising strategies for improving retention of nurses in hospital practice [6]. The literature review for this paper focused on papers which had used the MBI and/or NEO-FFI and targeted the general nursing population or nursing students.

### 1.2. Association of Demographics Characteristics with Burnout

Demographic variables such as age, education level, gender, and years of experience had been found to be significantly associated with emotional exhaustion (EE) (Table 1(a)). However, findings were inconsistent and it was difficult to compare findings across studies given different analysis methods. Most studies reported that younger nurses tend to score higher in EE compared to nurses in older age groups [7–12] while another study in Nigeria found that a greater proportion of older nurses experienced high EE as compared to younger nurses [13].

Marital status was also found to affect EE; however, findings were again inconsistent. Most studies found that nurses who were married were more prone to EE [9, 11, 12], while others reported that single participants scored significantly higher than the married participants [10, 13].

Females were found to suffer more EE than their male colleagues in studies from Shenyang, China [8], Japan [10], and Nigeria [13], whereas another study in Henan, China [9], reported that the male gender was positively associated with EE.

Data from Shenyang, China [8], suggested that senior-ranked nurses were more prone to EE while results from Nigeria and Shanghai, China, suggested that junior-ranked nurses were more at risk. The make-up of nurses in different ranks could have explained why some found higher EE risks among older nurses and junior-ranked ones, while others found higher EE risks among those younger and junior-ranked nurses.

Demographic characteristics such as age, gender, marital status, job rank, shift work, and experience have also been found to be at play on an individual's depersonalization level (Table 1(a)). Across several studies, it appears that older nurses tend to score higher in depersonalization [11–13]. However, Queiros et al.'s study found younger nurses to score higher in depersonalization [14]. Shift work appears to have an effect as well [11, 13, 15] though the extent of it is unclear.

Job rank has also been found to play a significant role in burnout, with literature suggesting that the higher an individual's rank, the higher his scores on personal accomplishment [13, 14, 16]. Age and experience have also been found to be significant and consistent factors [11, 13–16]; the older and more experienced an individual is, the higher his scores would be on personal accomplishment (Table 1(a)). Shift work, in particular night shifts [13], is purported to have a negative role in personal accomplishment [11, 15]. Though the number of studies that explored the relations between personal accomplishment and marital status is minimal, the results were consistent, reporting that unmarried individuals scored lower on personal accomplishment as compared to their married counterparts [11, 13].

### 1.3. Association of Personality with Burnout

Published literature on burnout and personality characteristics had suggested that certain personality traits could potentially act as a protective measure against burnout. For example, a strong sense of ability to control things that happen in life and at work was reported to protect nurses from EE, DP, and lack of PA [17]. Other traits such as conscientiousness are associated with an individual's persistency and self-discipline to get things done and hence such an individual is more likely to also enjoy higher personal accomplishment [18]. On the other hand, someone who tends to experience negative, distressing emotions is more likely to adopt maladaptive coping strategies and report feelings of emotional exhaustion [18].

Though many studies have explored various personality characteristics such as hardiness and anxiety and their relations with burnout, not many have explored the relations between the “Big Five” personality traits and the components of burnout. The Big Five model argues that each individual has five basic facets to their personality, namely, openness to experience, conscientiousness, extraversion, agreeableness, and neuroticism. Openness to experience is characterised by individuals who are highly reflective, sensitive, artistic, and imaginative [19]. Conscientiousness refers to individuals who are hard-working and self-disciplined and who possess traits in the similar vein. Extraversion is captured by the facets of warmth, gregariousness, assertiveness, activity, excitement seeking, and positive emotions. Agreeableness embodies empathy, love, friendliness, and cooperation; simply put, people who score high on agreeableness are the people you would describe as “nice.” Neuroticism is defined as an individual's experience of negative emotions, in other words, negative affectivity [19].

As summarised in Table 1(b), neuroticism was found to have a significant positive relationship with emotional exhaustion across all five studies reviewed [15, 16, 20–22]. Extraversion, on the other hand, was found to be negatively associated with emotional exhaustion [20, 21]. Takemura et al. [22] also reported a negative relationship between emotional exhaustion and extraversion, agreeableness, and conscientiousness. Though the results from these studies are significant, interpretations made on transferability of results should be done so with caution. In the case of the study by Takemura et al. [22], the population surveyed was nursing students while the study by Cañadas-De la Fuente et al. [20] employed a Spanish student sample and a version of the MBI that has been adapted and validated for a Spanish population.

A statistically significant association between depersonalization and extraversion was also identified [15, 20] with studies indicating that a more extraverted personality acts as a protective factor against depersonalization. An individual who scores high on extraversion is understood as one who is friendly, leads a life that is fast in pace, has the ability to converse at ease with strangers, and is able to thrive in environments that are noisy [19]. This finding was unsurprising given that the work of nurses involved being in a fast paced and noisy environment, being friendly, and providing comfort to people previously unknown to them. Neuroticism was identified to have a positive relation with depersonalization while openness and agreeableness were found to be associated with lower scores on depersonalization [16, 20, 22].

All five studies analysed in this review found a statistically significant positive correlation between extraversion and personal accomplishment [15, 16, 20–22]. While results were statistically significant for conscientiousness and personal accomplishment for the studies by Cañadas-De la Fuente et al. [20], Ganjeh et al. [15], and Watson et al. [21], the results by Cañadas-De la Fuente et al. [20] and Ganjeh et al. [15] demonstrated a positive one while Watson et al. [21] demonstrated a negative one. The study conducted by Watson et al. [21] was among nursing students, rather than working nurses, which could perhaps account for this difference. Openness and agreeableness [20] were also identified to have a positive linear relationship with personal accomplishment. Agreeableness is explained as individuals who are humble and modest and who have a tendency to note the achievements of others rather than theirs and would rather not appreciate attention to be brought upon them [19]. Again, nurses are generally understood as people who are happy when their patients are treated and discharged and work for the comfort of their patient rather than themselves, going so far as to put their patients before them, which could account for the relation between agreeableness and personal accomplishment.

## 2. Methods

### 2.1. Aims and Research Questions

The aims of this study were twofold. First, we sought to evaluate the prevalence and extent of burnout among nurses in Singapore. A second aim was to investigate the influence of demographic factors and personal characteristics on the burnout syndrome.

### 2.2. Design

A cross-sectional survey design was adopted.

### 2.3. Sample

All registered nurses working in Singapore General Hospital (a 1600-bedded tertiary acute care hospital in Singapore) were approached to participate in the study. Registered nurses included both enrolled nurses and staff nurses. In Singapore, enrolled nurses receive vocational training and support staff nurses in the management of patients. On the other hand, the entry qualification for nursing is a diploma for most staff nurses.

### 2.4. Instruments

#### 2.4.1. Sociodemographics Variables

Demographic details of gender, age, marital status, number of dependents, educational qualifications, number of years working as a nurse, shift patterns, clinical areas of work, and job grade were elicited using a self-administered questionnaire.

#### 2.4.2. Burnout

The experience of burnout was measured using the Maslach Burnout Inventory (MBI) [5]. It is the most widely used instrument which allowed the researchers to benchmark our findings with that of other countries. The MBI consisted of 22 items, which measured 3 components of burnout, namely, emotional exhaustion (EE), depersonalization (DP), and personal accomplishment (PA). Each item could be answered on a 7-point Likert scale ranging from “never” (=0) to “daily” (=6). Burnout is indicated by high scores on emotional exhaustion and depersonalization and low scores on personal accomplishment. It was reported that reliability (as measured by Cronbach alpha) was 0.9 for the emotional exhaustion scale, 0.79 for the depersonalization scale, and 0.71 for the personal accomplishment scale [24]. The MBI also correlated well with other burnout scales such as the Shirom Melamed Burnout Questionnaire [25]. The originators of the instrument did not specify a clinical threshold that indicates the presence or absence of burnout. Some studies have used extreme quartiles of the MBI score [26]. Similarly, for this study, when scores for emotional exhaustion and depersonalization were above the 3rd quartile and scores for personal accomplishment were below the 1st quartile, the individual is classified as being burnt out.

#### 2.4.3. Personality

Personality characteristics were measured using the NEO Five-Factor Inventory (NEO-FFI). It consisted of 60 items, rated on a five-point scale (strongly disagree, disagree, neutral, agree, and strongly agree), which measured the five personality dimensions of neuroticism, extraversion, openness, agreeableness, and conscientiousness. The coefficient reliability estimates were 0.83 (neuroticism), 0.75 (extraversion), 0.68 (openness), 0.73 (agreeableness), and 0.79 (conscientiousness) [17].

### 2.5. Data Collection

Potential participants were informed of the study via staff meetings. The questionnaires and participant information sheets containing the informed consent forms for participants to sign were delivered to the clinical areas. Participants would complete the questionnaire upon signing of informed consent. To ensure confidentiality and anonymity, consent forms and questionnaires were collected separately. Email reminders were sent out 2 weeks after to encourage participation. Participants also received a token of appreciation (pocket calculator) upon returning the questionnaires.

### 2.6. Data Analysis

Frequencies and percentages were used to describe demographic and personality variables in the study dataset within each component of burnout measured using the Maslach Burnout Inventory (MBI), namely, emotional exhaustion (EE), depersonalization (DP), and personal accomplishment (PA). All of the components of MBI were categorized into low, average, and high values using the specified cut-off points for the medicine subgroup [27]. Respondents were also categorized according to their scores for each personality domain. All of the five personality dimensions measured using the NEO Five-Factor Inventory (NEO-FFI) of neuroticism, extraversion, openness, agreeableness, and conscientiousness were recoded according to the specified cut-off points (ref) into 5 categories: very low, low, average, high, and very high. Number of negative personality traits (in relation to higher risk of burnout) was also calculated for each respondent. For example, if an individual scored high on neuroticism and low on extraversion, openness, agreeableness, and conscientiousness, he/she will be deemed to have 5 negative traits.

Univariable and multivariable logistic regression models were used to investigate associations between demographic variables, personality dimensions, and components of the burnout syndrome (EE, DP, and PA). Logistic regression was also used to assess the effect of the composite variable and number of negative personality dimensions on the components of the burnout syndrome. Multivariable logistic regression identified the least number of demographic variables and personality dimensions that are significantly associated with each of the 3 components of the burnout syndrome. The effects of these variables are expressed as odds-ratios and associated 95% confidence intervals.

All statistics were performed with SPSS 21.0 software. *p* values less than 0.05 are considered as statistically significant.

## 3. Results

Among 3588 nurses who were invited to participate, 1830 responded (response rate: 51%). Due to missing data, results from 1826 respondents were used in the analysis. Most of the respondents were female (93%), were less than or equal to 39 years of age (76%), and had worked in the current hospital for less than 10 years (74%). Most were Chinese (43%) and 41% had a bachelor or postgraduate degree. Majority was staff nurses (68%) and reported performing 3 shifts (74%). Using the NEO Five-Factor Inventory (NEO-FFI), most nurses were low or average in neuroticism (72%) and average or high in extraversion (82%), openness (79%), agreeableness (77%), and conscientiousness (81%). The MBI identified 39% of the respondents to have high EE (cut-off score of ≥27), 40% having high DP (cut-off score of ≥10), and 59% having low PA (cut-off score of ≤33), according to the specified cut-off points for the medicine subgroup. The mean score for EE was 23.5 (SD = 11.8), for DP was 8.6 (SD = 5.9), and for PA was 30.9 (SD = 8.6), for the three dimensions of burnout, measured in the continuous scale. Tables 2(a) and 2(b) present the number of nurses and the percentages that represent the prevalence of the levels of burnout against the respective demographic and personality factors.

Table 3 presents the associations between the demographic variables and personality dimensions on the risk of burnout, using univariate logistic regression. Males were 1.58 times more likely to experience high depersonalization compared to females (95% CI: 1.10–2.27). Nurses aged 30 years or over were less likely to experience high EE and high DP (*p* < 0.05) compared to those who are less than 30 years of age. Nurses aged 60 years or older were about 4.2 times less likely to experience low PA compared to nurses who were less than 30 years old (OR = 0.24; 95% CI: 0.12–0.51). Race was significantly associated with high EE (*p* = 0.01), high DP (*p* < 0.0005), and low PA (*p* < 0.0005). Staff working 10 years or more were less likely to experience high EE (*p* < 0.05), high DP (*p* < 0.0005), and low PA (*p* < 0.05).

Nurses with diploma and advanced diploma educational qualification were more likely to experience high EE (*p* < 0.01) and high DP (*p* < 0.01) compared to nurses who only had vocational training, the base level to be credited as a registered nurse in Singapore. Nurses with a bachelor or postgraduate degree were about 2 times less likely to have low PA (OR = 0.52; 95% CI: 0.40–0.68), compared to nurses with vocational training. Staff nurses were more likely to experience high EE (*p* < 0.0005), high DP (*p* < 0.0005), and low PA (*p* < 0.05) compared to enrolled nurses. Nursing officers were also less likely to have low PA compared to enrolled nurses (*p* < 0.0005). Nurses who worked 3 shift patterns were 1.52 times more likely to experience high EE (*p* = 0.004) and 1.76 times more likely to experience high DP (*p* < 0.0005).

Nurses with high and very high neuroticism were 3.93 times more likely to experience high EE, 3.44 times more likely to experience high DP, and 2.38 times more likely to experience low PA (all *p* < 0.0005), compared to nurses who had average or below average neuroticism. Nursing staff with low or very low extraversion, agreeableness, and conscientiousness were more likely to experience high EE, high DP, and low PA (all *p* = 0.0005), compared to nurses with average and above average values on the respective personality dimension.

On amalgamating the 5 personality dimensions to a single composite variable representing the number of negative personality traits, a very significant association (*p* < 0.0005) was found between increasing number of negative personality traits and high EE, high DP, and low PA.

Multivariable logistic regression to assess the association between the demographic variables and personality dimensions on the components of burnout is presented in Table 4. It also assisted in identifying the least number of significant variables predicting high EE, high DP, and low PA. Variables predicting high EE were age, job grade, dependents, neuroticism, extraversion, and agreeableness. High DP was associated with gender, age, race, job grade, dependents, neuroticism, and agreeableness. Variables predicting low PA were age, race, job grade, neuroticism, extraversion, openness, and conscientiousness.

The variables age, job grade, and neuroticism were significantly associated with each of the 3 components of the MBI, in the multivariable models.

## 4. Discussion

This study indicated that the nurses in Singapore experienced comparable levels of EE and higher levels of DP but higher levels of PA than nurses in China [28] whereby mean EE levels of 23.9, mean DP levels of 7.9, and mean PA levels of 27.5 were reported. On the other hand, nurses in Singapore experienced lower levels of EE and DP and higher levels of PA than nurses in UK. Mean EE scores of 25.6, DP scores of 16.5, and PA scores of 15.6 were reported by a study on nurses from 3 NHS trusts in UK [29].

Even though many previous studies used MBI as a measurement tool for burnout, it was difficult to compare prevalence rates across studies due to use of different cut-off points [23, 30, 31] to identify nurses at high risk of burnout for each of the 3 components. In a study of registered nurses across 12 European countries [30], lower percentages of nurses with high EE (27%), high DP (10%), and reduced PA (17%) were reported as compared to results for the current study (EE 38%, DP 38%, and reduced PA 58%). However, in the European study, cut-off points for overall population were used to analyse the data, instead of cut-off points for the medicine subgroup (i.e., a higher cut-off score was used for high DP, and a lower cut-off score was used for reduced PA) [27].

In congruence with most of previous studies' findings [7–12, 32], we found that older nurses, with more years of working experience, are less likely to experience burnout as compared to younger nurses. This could be because, with more experience, older nurses could cope better with job demands. Similar to other studies [11], we also found that shift work (in particular 3 shifts) has a detrimental effect on the experience of burnout. This could be related to poor sleep quality in relation to having to perform rotating shifts, which in turn resulted in higher experience of burnout [33].

Our study also suggested that job grade has a significant influence on all 3 dimensions of burnout. Staff nurses tend to experience higher levels of EE and DP but also higher PA than enrolled nurses. This could be because staff nurses have more roles and responsibilities than enrolled nurses but at the same time enjoyed higher academic qualifications.

In our study, male nurses were found to be more likely to experience high levels of DP, but not in other dimensions of burnout. This is in contrast to findings from previous studies [10, 13], whereby male nurses tend to experience lower burnout levels. We also found that race was significantly associated with the experience of DP and PA. Controlling for other factors, nurses of Indian and Filipino ethnic groups tend to experience less DP and PA than Chinese nurses. This could be due to differences in general cultural beliefs and attitudes.

Our study confirmed the role of personality traits in influencing the experience of burnout. Strong associations were found between different personality traits and all 3 dimensions of burnout. Consistent with that of previous studies [15, 16, 20, 22], we also found that high scores on extraversion, openness, agreeableness, and conscientious had a protective effect on burnout, while high scores on neuroticism exerted a detrimental effect. The more is the number of negative personality traits that a nurse possessed, the greater is his/her risk of experiencing burnout. On a personal front, individuals who score high on neuroticism tend to exaggerate the severity and frequency of personal problems that they face [16] and employ coping mechanisms that require them to avoid or distract themselves from the problem, subsequently leading them to experience and score high in emotional exhaustion and depersonalization and low in personal accomplishment [20]. Extraverted individuals are synonymous with optimists, voicing out frustrations lesser in comparison and adopting a positive outlook towards their working situation [16, 20]. Extraverted individuals can also be expected to perform well in a work environment such as nursing which demands a high aptitude of social skills [15, 20], suggesting that such a trait could be protective against burnout. Individuals who score high on openness tend to embrace and succeed in new and vague situations [20]; individuals who are open to new experiences are willing to learn and view challenges as opportunities to do so, magnifying their personal accomplishments from their viewpoint and minimising their emotional exhaustion in that process [16]. Agreeable individuals are flexible, willing to cooperate, warm, and sympathetic [15, 20]. These individuals are also responsible, planning their work and time in an efficient manner, and thus utilise coping strategies that focus directly on the problem [20]. Highly agreeable individuals have an innate tendency to care and nurture, suggesting that they would report lower levels of emotional exhaustion and depersonalization [16]. Since individuals who score high on agreeableness are willing to nurture, nurses who improve the overall comfort of their patients might view that as their personal accomplishments, subsequently reporting a higher level of it [16]. Conscientiousness is the most desired trait in job performance [15], with individuals exhibiting hard-work, perseverance, and a thirst for achievement [16] which would boost their level of personal accomplishments. These individuals make use of active coping mechanisms, which has been identified to be associated with lower health complaints and, in turn, lower emotional exhaustion [16].

Given the current study results, it is important that health care organizations give weight to the need for prevention programmes (which can include aspects of positive psychology and training in coping strategies), targeted at nurses with vulnerable personality traits [34]. Nurses at risk may benefit from such programmes to improve their coping skills in dealing with stressful work situations and in reducing their negative emotional responses under such circumstances [18].

This study has limitations that should be taken into account when interpreting the results. A cross-sectional survey design was adopted and hence results only demonstrate association and not causal relationships. Nurses were only recruited from one acute care hospital, and hence results might not be generalizable to other settings such as community and primary care.

## 5. Conclusion

Overall, this study contributed to the understanding of burnout among nurses in Singapore and confirmed the role of personality traits in the experience of burnout. Our findings would help to inform strategies by nurse leaders and health care administrators working in a tertiary care hospital, Asian context. Results of the current study suggested that programmes to prevent the onset of burnout should be targeted at younger staff nurses. Administrators could also consider the usefulness of personality profiling, in order to direct resources to prevent burnout among nurses with vulnerable personality traits.

## Figures and Tables

**(a):** 

Demographic factors significantly associated with emotional exhaustion							
	Wu et al., 2014 [7]	Li et al., 2014 [8]	Yao et al., 2013 [9]	Lasebikan and Oyetunde, 2012 [13]	Ohue et al., 2011 [10]	Xie et al., 2011 [11]	Al-Turki et al., 2010 [12]
Age	✓	✓	✓	✓	✓	✓	✓
Gender		✓	✓	✓	✓		
Marital status			✓	✓	✓	✓	✓
Job rank		✓		✓		✓	
Experience		✓				✓	✓
Education	✓						
Being a foreign nurse							✓

Demographic factors significantly associated with depersonalization							
	Queiros et al., 2013 [14]		Lasebikan and Oyetunde, 2012 [13]		Xie et al., 2011 [11]		Al-Turki et al., 2010 [12]

Age	✓		✓		✓		✓
Gender	✓		✓				
Marital status			✓		✓		
Job rank			✓		✓		
Experience					✓		
Shift work			✓		✓		
Being a foreign nurse							✓
Type of ward							✓

Demographic factors significantly associated with personal accomplishment							
	Queiros et al., 2013 [14]		Lasebikan and Oyetunde, 2012 [13]		Lin et al., 2009 [23]		Xie et al., 2011 [11]

Age			✓		✓		✓
Gender			✓				
Marital status			✓				✓
Job rank	✓		✓		✓		
Experience	✓				✓		✓
Shift work			✓				✓

**(b):** 

Personality factors significantly associated with burnout					
	Cañadas-De la Fuente et al., 2015 [20]	Ganjeh et al., 2009 [15]	Zellars et al., 2000 [16]	Watson et al., 2008 [21]	Takemura et al., 2015 [22]
Openness↑	↓EE				
	↓DP		↓DP	↓DP	
	↑PA		↑PA	↑PA	↑PA

Conscientiousness↑	↓EE			↓EE	↓EE
	↓DP			↓DP	↓DP
	↑PA	↑PA		↑PA	

Extraversion↑	↓EE	↓EE		↓EE	↓EE
	↓DP		↓DP	↓DP	↓DP
	↑PA	↑PA	↑PA	↑PA	↑PA

Agreeableness↑	↓EE	↓DP	↓DP	↓DP	↓EE
	↓DP				↓DP
	↑PA	↑PA			↑PA

Neuroticism↑	↑EE	↑EE	↑EE	↑EE	↑EE
	↑DP	↑DP		↑DP	↑DP
	↓PA				↓PA

**Table:** (a) Participants' demographics and prevalence of burnout

		Emotional exhaustion		Depersonalization		Personal accomplishment	
		Low/average *N* (%)	High *N* (%)^*∗*^	Low/average *N* (%)	High *N* (%)^*∗*^	Average/high *N* (%)	Low *N* (%)^*∗*^
Gender	Female	1002 (61.5)	627 (38.5)	998 (61.3)	631 (38.7)	663 (40.7)	966 (59.3)
	Male	72 (57.1)	54 (42.9)	63 (50)	63 (50)	53 (42.1)	73 (57.9)

Age (years)	≤29	512 (55.4)	413 (44.6)	489 (52.9)	436 (47.1)	357 (38.6)	568 (61.4)
	30–39	241 (61.6)	150 (38.4)	240 (61.4)	151 (38.6)	153 (39.1)	238 (60.9)
	40–49	179 (75.2)	59 (24.8)	181 (76.1)	57 (23.9)	106 (44.5)	132 (55.5)
	50–59	96 (68.6)	44 (31.4)	102 (72.9)	38 (27.1)	62 (44.3)	78 (55.7)
	≥60	31 (86.1)	5 (13.9)	33 (91.7)	3 (8.3)	26 (72.2)	10 (27.8)

Race	Chinese	456 (59.1)	316 (40.9)	430 (55.7)	342 (44.3)	320 (41.5)	452 (58.5)
	Malay	194 (56.2)	151 (43.8)	190 (55.1)	155 (44.9)	113 (32.8)	232 (67.2)
	Indian	154 (68.1)	72 (31.9)	151 (66.8)	75 (33.2)	84 (37.2)	142 (62.8)
	Filipino	178 (66.9)	88 (33.1)	188 (70.7)	78 (29.3)	139 (52.3)	127 (47.7)
	Others	101 (62.3)	61 (37.7)	112 (69.6)	49 (30.4)	65 (40.1)	97 (59.9)

Highest qualification	Vocational training	245 (67.7)	117 (32.3)	245 (67.7)	117 (32.3)	120 (33.1)	242 (66.9)
	Diploma	263 (56)	207 (44)	248 (52.8)	222 (47.2)	166 (35.3)	304 (64.7)
	Advanced diploma	101 (54.3)	85 (45.7)	104 (55.9)	82 (44.1)	71 (38.2)	115 (61.8)
	Bachelor/postgraduate	449 (63.5)	258 (36.5)	447 (63.2)	260 (36.8)	344 (48.7)	363 (51.3)

Job grade	Enrolled nurse	290 (69.5)	127 (30.5)	283 (68)	133 (32)	145 (34.8)	272 (65.2)
	Staff nurse	689 (57.1)	518 (42.9)	684 (56.7)	523 (43.3)	496 (41.1)	711 (58.9)
	Nursing officer	96 (72.2)	37 (27.8)	96 (72.2)	37 (27.8)	75 (56.4)	58 (43.6)

Shift patterns	Fixed hours	171 (67.9)	81 (32.1)	176 (70.1)	75 (29.9)	117 (46.4)	135 (53.6)
	2 shifts	80 (72.1)	31 (27.9)	76 (68.5)	35 (31.5)	44 (39.6)	67 (60.4)
	3 shifts	741 (58.2)	532 (41.8)	727 (57.1)	546 (42.9)	512 (40.2)	761 (59.8)
	Permanent night	73 (73.7)	26 (26.3)	71 (71.7)	28 (28.3)	37 (37.4)	62 (62.6)

Years working in current hospital	<10	739 (57.9)	537 (42.1)	714 (56)	562 (44)	489 (38.3)	787 (61.7)
	10–19	119 (65.7)	62 (34.3)	129 (71.3)	52 (28.7)	82 (45.3)	99 (54.7)
	20–29	131 (72)	51 (28)	133 (73.1)	49 (26.9)	90 (49.5)	92 (50.5)
	≥30	78 (75.7)	25 (24.3)	80 (77.7)	23 (22.3)	50 (48.5)	53 (51.5)

Dependents	No	253 (63.1)	148 (36.9)	257 (64.1)	144 (35.9)	155 (38.7)	246 (61.3)
	Yes	818 (60.5)	533 (39.5)	804 (59.6)	546 (40.4)	561 (41.5)	790 (58.5)

**Table:** (b) Participants' personality and prevalence of burnout

		Emotional exhaustion		Depersonalization		Personal accomplishment	
		Low/average *N* (%)	High *N* (%)^*∗*^	Low/average *N* (%)	High *N* (%)^*∗*^	Average/high *N* (%)	Low *N* (%)^*∗*^
Neuroticism^#^	VL, L, A	875 (70.5)	366 (29.5)	856 (69)	385 (31)	576 (46.4)	665 (53.6)
	H, VH	187 (37.9)	307 (62.1)	194 (39.3)	300 (60.7)	132 (26.7)	362 (73.3)

Extraversion^#^	A, H, VH	922 (64.6)	506 (35.4)	897 (62.8)	531 (37.2)	650 (45.5)	778 (54.5)
	L, VL	140 (45.6)	167 (54.4)	153 (49.8)	154 (50.2)	58 (18.9)	249 (81.1)

Openness^#^	A, H, VH	839 (61.3)	529 (38.7)	843 (61.6)	525 (38.4)	609 (44.5)	759 (55.5)
	L, VL	223 (60.8)	144 (39.2)	207 (56.4)	160 (43.6)	99 (27)	268 (73)

Agreeableness^#^	A, H, VH	865 (64.9)	468 (35.1)	871 (65.3)	462 (34.7)	583 (43.7)	750 (56.3)
	L, VL	197 (49)	205 (51)	179 (44.5)	223 (55.5)	125 (31.1)	277 (68.9)

Conscientiousness^#^	A, H, VH	903 (63.8)	513 (36.2)	906 (64)	510 (36)	636 (44.9)	780 (55.1)
	L, VL	159 (49.8)	160 (50.2)	144 (45.1)	175 (54.9)	72 (22.6)	247 (77.4)

Number of negative personality traits	0	524 (73)	194 (27)	533 (74.2)	185 (25.8)	395 (55)	323 (45)
	1	296 (60.9)	190 (39.1)	283 (58.2)	203 (41.8)	196 (40.3)	290 (59.7)
	2	148 (50.9)	143 (49.1)	144 (49.5)	147 (50.5)	74 (25.4)	217 (74.6)
	3	66 (42.9)	88 (57.1)	61 (39.6)	93 (60.4)	31 (20.1)	123 (79.9)
	≥4	28 (32.6)	58 (67.4)	29 (33.7)	57 (66.3)	12 (14)	74 (86)

^*∗*^Higher risk of burnout.

^#^Categories for personality factors relative to risk of burnout: VL: very low; L: low; A: average; H: high; VH: very high.

**Table 3:** Association between demographic factors and personality factors with burnout using univariate logistic regression. Effects are presented as odds-ratio and associated 95% confidence intervals.

		High emotional exhaustion		High depersonalization		Low personal accomplishment	
		Univariate OR, 95% CI	*p* value	Univariate OR, 95% CI	*p* value	Univariate OR, 95% CI	*p* value
Gender	Female	1		1		1	
	Male	1.20 (0.83–1.73)	0.333	1.58 (1.10–2.27)	**0.013**	0.95 (0.66–1.37)	0.764

Age (years)	Overall		<0.0005		<0.0005		0.002
	≤29	1		1		1	
	30–39	0.77 (0.61–0.98)	**0.035**	0.71 (0.55–0.90)	**0.005**	0.98 (0.77–1.25)	0.855
	40–49	0.41 (0.30–0.56)	<**0.0005**	0.35 (0.26–0.49)	<**0.0005**	0.78 (0.59–1.04)	0.095
	50–59	0.57 (0.39–0.83)	**0.004**	0.42 (0.28–0.62)	<**0.0005**	0.79 (0.55–1.13)	0.200
	≥60	0.20 (0.08–0.52)	**0.001**	0.10 (0.03–0.34)	<**0.0005**	0.24 (0.12–0.51)	**<0.0005**

Race	Overall		0.010		<0.0005		<0.0005
	Chinese	1		1		1	
	Malay	1.12 (0.87–1.45)	0.375	1.03 (0.80–1.32)	0.846	1.45 (1.11–1.90)	**0.006**
	Indian	0.68 (0.49–0.92)	**0.014**	0.62 (0.46–0.85)	**0.003**	1.20 (0.88–1.62)	0.249
	Filipino	0.71 (0.53–0.96)	**0.024**	0.52 (0.39–0.70)	<**0.0005**	0.65 (0.49–0.86)	**0.002**
	Others	0.87 (0.62–1.24)	0.440	0.55 (0.38–0.79)	**0.001**	1.06 (0.75–1.49)	0.755

Highest qualifications	Overall		0.001		<0.0005		<0.0005
	Vocational training	1		1		1	
	Diploma	1.65 (1.24–2.19)	**0.001**	1.87 (1.41–2.49)	**<0.0005**	0.91 (0.68–1.21)	0.514
	Advanced diploma	1.76 (1.23–2.53)	**0.002**	1.65 (1.15–2.38)	**0.007**	0.80 (0.56–1.16)	0.243
	Bachelor/postgraduate	1.20 (0.92–1.57)	0.176	1.22 (0.93–1.59)	0.149	0.52 (0.40–0.68)	**<0.0005**

Job grade	Overall		<0.0005		<0.0005		<0.0005
	Enrolled nurse	1		1		1	
	Staff nurse	1.72 (1.35–2.18)	**<0.0005**	1.63 (1.29–2.06)	<**0.0005**	0.76 (0.61–0.96)	**0.023**
	Nursing officer	0.88 (0.57–1.36)	0.563	0.82 (0.53–1.26)	0.368	0.41 (0.28–0.61)	**<0.0005**

Shift patterns	Overall		<0.0005		<0.0005		0.262
	Fixed hours	1		1		1	
	2 shifts	0.82 (0.50–1.34)	0.423	1.08 (0.67–1.75)	0.753	1.32 (0.84–2.08)	0.231
	3 shifts	1.52 (1.14–2.02)	**0.004**	1.76 (1.32–2.36)	**<0.0005**	1.29 (0.98–1.69)	0.068
	Permanent night	0.75 (0.45–1.27)	0.282	0.93 (0.55–1.55)	0.768	1.45 (0.90–2.34)	0.125

Years working in current hospital	Overall		<0.0005		<0.0005		0.005
	<10	1		1		1	
	10–19	0.72 (0.52–0.99)	**0.046**	0.51 (0.36 –0.72)	**<0.0005**	0.75 (0.55–1.03)	0.072
	20–29	0.54 (0.38–0.75)	**<0.0005**	0.47 (0.33 –0.66)	**<0.0005**	0.64 (0.47–0.87)	**0.004**
	≥30	0.44 (0.28–0.70)	**0.001**	0.37 (0.23–0.59)	**<0.0005**	0.66 (0.44–0.99)	**0.042**

Dependents	No	1		1		1	
	Yes	1.11 (0.89–1.40)	0.359	1.21 (0.96–1.53)	0.103	0.89 (0.71–1.12)	0.304

Neuroticism	VL, L, A	1		1		1	
	H, VH	3.93 (3.15–4.89)	**<0.0005**	3.44 (2.77–4.27)	**<0.0005**	2.38 (1.89–2.99)	**<0.0005**

Extraversion	A, H, VH	1		1		1	
	L, VL	2.17 (1.69–2.79)	**<0.0005**	1.70 (1.33–2.18)	**<0.0005**	3.59 (2.65–4.86)	**<0.0005**

Openness	A, H, VH	1		1		1	
	L, VL	1.02 (0.81–1.30)	0.843	1.24 (0.98–1.57)	0.070	2.17 (1.69–2.80)	**<0.0005**

Agreeableness	A, H, VH	1		1		1	
	L, VL	1.92 (1.54–2.41)	**<0.0005**	2.35 (1.87–2.95)	**<0.0005**	1.72 (1.36–2.18)	**<0.0005**

Conscientiousness	A, H, VH	1		1		1	
	L, VL	1.77 (1.39–2.26)	**<0.0005**	2.16 (1.69–2.76)	**<0.0005**	2.80 (2.11–3.71)	**<0.0005**

Number of negative personality traits	Overall		<0.0005		<0.0005		<0.0005
	0	1		1		1	
	1	1.73 (1.36–2.22)	**<0.0005**	2.07 (1.62–2.64)	**<0.0005**	1.81 (1.43–2.29)	**<0.0005**
	2	2.61 (1.97–3.46)	**<0.0005**	2.94 (2.21–3.91)	**<0.0005**	3.59 (2.65–4.85)	**<0.0005**
	3	3.60 (2.52–5.16)	**<0.0005**	4.39 (3.05–6.32)	**<0.0005**	4.85 (3.19–7.39)	**<0.0005**
	≥4	5.60 (3.46–9.04)	**<0.0005**	5.66 (3.51–9.13)	**<0.0005**	7.54 (4.03–14.12)	**<0.0005**

**Table 4:** Association between demographic factors and personality factors with burnout using multivariable logistic regression. Effects are presented as odds-ratio and associated 95% confidence intervals.

		High emotional exhaustion		High depersonalization		Low personal accomplishment	
		Multivariable OR, 95% CI	*p* value	Multivariable OR, 95% CI	*p* value	Multivariable OR, 95% CI	*p* value
Gender	Female			1			
	Male			**1.85 (1.21–2.81)**	**0.004**		

Age (years)	Overall		0.002		<0.0005		0.023
	≤29	1		1		1	
	30–39	0.76 (0.57–1.01)	0.058	**0.70 (0.53–0.94)**	**0.017**	**1.37 (1.03–1.82)**	**0.028**
	40–49	**0.51 (0.35–0.74)**	**<0.0005**	**0.39 (0.27–0.57)**	<**0.0005**	0.95 (0.68–1.320)	0.752
	50–59	0.80 (0.52–1.25)	0.336	**0.45 (0.29–0.70)**	<**0.0005**	1.06 (0.70–1.61)	0.774
	≥60	**0.26 (0.09–0.78)**	**0.016**	**0.13 (0.04–0.43)**	**0.001**	**0.39 (0.16–0.91)**	**0.030**

Race	Overall				<0.0005		0.006
	Chinese			1		1	
	Malay			0.97 (0.71–1.31)	0.829	1.20 (0.89–1.63)	0.237
	Indian			**0.62 (0.43–0.90)**	**0.011**	1.11 (0.78–1.56)	0.571
	Filipino			**0.50 (0.35–0.72)**	<**0.0005**	**0.62 (0.45–0.86)**	**0.004**
	Others			**0.51 (0.33–0.79)**	**0.002**	1.11 (0.74–1.66)	0.617

Highest qualifications	Overall						
	Vocational training						
	Diploma						
	Advanced diploma						
	Bachelor/postgraduate						

Job grade	Overall		0.001		0.005		<0.0005
	Enrolled nurse	1		1		1	
	Staff nurse	**1.66 (1.26–2.18)**	<**0.0005**	**1.55 (1.17–2.06)**	**0.002**	**0.67 (0.51–0.88)**	**0.004**
	Nursing officer	1.11 (0.66–1.89)	0.687	1.07 (0.63–1.82)	0.807	**0.37 (0.23–0.60)**	**<0.0005**

Shift patterns	Overall		0.039				
	Fixed hours	1					
	2 shifts	0.68 (0.39–1.19)	0.173				
	3 shifts	1.18 (0.83–1.67)	0.360				
	Permanent night	0.70 (0.39–1.27)	0.246				

Years working in current hospital	Overall						
	<10						
	10–19						
	20–29						
	≥30						

Dependents	No	1		1			
	Yes	**1.37 (1.05–1.79)**	**0.022**	**1.80 (1.37–2.37)**	**<0.0005**		

Neuroticism	VL, L, A	1		1		1	
	H, VH	**3.24 (2.54–4.13)**	**<0.0005**	**2.79 (2.20–3.55)**	**<0.0005**	**1.79 (1.39–2.32)**	**<0.0005**

Extraversion	A, H, VH	1				1	
	L, VL	**1.64 (1.23–2.19)**	**0.001**			**2.80 (1.98–3.95)**	**<0.0005**

Openness	A, H, VH					1	
	L, VL					**1.66 (1.25–2.21)**	**<0.0005**

Agreeableness	A, H, VH	1		1			
	L, VL	**1.46 (1.12–1.89)**	**0.004**	**1.80** ** (1.39–2.34)**	**<0.0005**		

Conscientiousness	A, H, VH					1	
	L, VL					**1.76 (1.28–2.41)**	**0.001**

Blank cells indicate that the association was not significant: *p* > 0.05.
